# First description of the sexuals of *Camponotus opaciventris* Mayr, 1879 (Hymenoptera, Formicidae), with notes on distribution in Western Himalaya

**DOI:** 10.3897/BDJ.4.e10464

**Published:** 2016-12-08

**Authors:** Aijaz Ahmad Wachkoo, Shahid Ali Akbar

**Affiliations:** ‡ University of Kashmir, Srinagar, India; § Central Institute of Temperate Horticulture, Srinagar, India

**Keywords:** Ants, Himalaya, redescription, distribution, taxonomy.

## Abstract

**Background:**

The taxonomy of *Camponotus* ants in India is mostly based on the worker caste, described in about 96% of the known species (AntWeb 2016). However, nearly 48% of these ant species are only known from workers, with no record of sexual forms. To improve knowledge of Indian *Camponotus*, we here describe sexuals of *Camponotus opaciventris* Mayr 1879.

**New information:**

The hitherto unknown sexuals of *Camponotus opaciventris* Mayr 1879 are described for the first time. Workers are redescribed and distribution of this ant species in Indian Western Himalaya is herewith detailed.

## Introduction

*Camponotus* Mayr (1861) is the world’s largest and widespread genus of ants found in all biogeographical regions (Hölldobler and Wilson 1990, AntWeb 2016). It is currently represented by 1,099 species and 495 subspecies, supplemented by 32 fossil species (AntWeb 2016). In India this genus is represented by 83 species and subspecies (AntWeb 2016, Bharti et al. 2016), however the status of many species and subspecies remains dubious and unclear (Bharti and Wachkoo 2014, Bharti and Wachkoo 2015, Bharti et al. 2016).

*Camponotus opaciventris* has a history of taxonomic confusion (Dietrich 2004); it was described in 1879 and later was synonomized with *Camponotus sericeus* (Fabricius 1798) by Bingham (1903). Subsequently Forel (1892), Forel (1908) treated it as infraspecific taxon of *C. sericeus* whilst Emery (1896), Emery (1925), Radchenko (1996) followed this view. Most recently, Dietrich (2004) revived *C. opaciventris* from synonymy raised it back to species status. *Camponotus opaciventris* is well represented in collections from Indian Western Himalayas, but has been frequently misidentified as widespread *C. sericeus* (Wachkoo 2013).

Western Himalayan region in India is represented by the states of Jammu and Kashmir in the west, Himachal Pradesh in the middle and Uttarakhand in the east. Faunal diversity of Western Himalaya is rich and diversified due to varied climatic conditions ranging from tropical in Shivalik foothills to very cold environment in the Trans Himalaya. There is a dominance of Palaearctic and endemic fauna above timber line (3000 m) and Oriental and some Palaearctic and Ethiopian elements at lower and middle altitudes (Sidhu et al. 2012, Shah et al. 2014, Wachkoo et al. 2016).

On examining our material collected from Western Himalayas and Western Ghats we find no evidence for the presence of *C. sericeus* in the Western Himalayas. For this reason and hitherto unknown sexuals we redescribe the worker caste and describe the queen and male castes for the first time. Images of all castes and a worker based comparative diagnosis are provided to clarify the species boundaries of *C. opaciventris* and *C. sericeus*. Information on the distribution and ecology of this species are also given.

## Materials and methods

The specimens were obtained by visual searching and hand-collecting. The morphological study was conducted on a Nikon SMZ 1500 stereo zoom microscope. For digital images, an Evolution MP digital camera was used on the same microscope with Auto-Montage (Syncroscopy, a division of Synoptics Ltd.) software. The images were processed with Adobe Photoshop CS5. Specimens examined for this study are deposited in PUPAC, Punjabi University Patiala Ant Collection and NHMW, Natural History Museum, Vienna, Austria. Some worker specimens will be deposited in BMNH, Natural History Museum, London, U.K. and CASC, California Academy of Sciences, San Francisco, United States of America. Morphological terminology for genitalia follow (Boudinot 2013); measurements, in millimeters, and indices follow (Wachkoo 2015) and are provided below.

HL - Maximum length of head in full-face view, measured in straight line from the anteriormost point of the median clypeal margin to the midpoint of the posterior head margin.

HW - Maximum head width in full-face view, excluding the portion of eyes that extends past the lateral margins of the head.

EL - Maximum eye length as measured with the head oriented obliquely to show full surface of eye.

SL - Maximum length of the scape excluding the basal neck and condyle.

PnW - Maximum width of pronotum in dorsal view.

ML - Mesosomal length in profile, from the anteriormost border of the pronotum, excluding the pronotal cervix to the posterior basal angle of the metapleuron.

MTL - Maximum length of the mesotibia with full width and length positioned in visual plane, measured from the most distal point near the extensor profile to the proximal constriction point of flexor profile.

HTL - Maximum length of the metatibia with full width and length positioned in visual plane, measured from the most distal point near the extensor profile to the proximal constriction point of flexor profile.

PL - Maximum length of the petiole in profile, measured in a straight horizontal line from immediately above the dorsal base of the anterior petiolar tubercle to the posterior margin.

GL - Length of the gaster in profile from the anteriormost point of the first gastral segment to the posteriormost point.

TL - Total length: HL+ML+PL+GL.

CI - Cephalic index: HW/HL × 100.

SI - Scape index: SL/HW × 100.

REL - Relative eye length index: EL/HL × 100.

## Taxon treatments

### Camponotus opaciventris

Mayr, 1879

Camponotus opaciventris Mayr 1879: 648 (w.) Syntype workers: Kolkata, India [NHMW]. [*Camponotus opaciventris*Smith 1873: viii. Nomen nudum, attributed to Mayr]. Combination in Camponotus (Orthonotomyrmex): Emery 1925: 126. Junior synonym of *Camponotus sericeus*: Bingham 1903: 376. Subspecies of *Camponotus sericeus*: Forel 1892: 223; Emery 1896: 376; Forel 1908: 6; Emery 1925: 126; Radchenko 1996: 1197. Raised to species: Dietrich 2004: 326; here confirmed.

#### Materials

- **Type status:** Syntype. **Occurrence:** recordedBy: Rothney; sex: worker; **Taxon:** scientificNameID: C. opaciventris; **Location:** country: India; stateProvince: West Bengal; locality: Kolkata; **Record Level:** institutionCode: NHMW
- **Type status:** Other material. **Occurrence:** recordedBy: Aijaz A. Wachkoo; individualCount: 2; sex: worker; **Taxon:** scientificNameID: C. opaciventris; **Location:** country: India; stateProvince: Himachal Pradesh; locality: Andretta; verbatimElevation: 940 m; verbatimCoordinates: 32.0744°N, 76.5856°E; **Event:** eventDate: 11/06/2010; **Record Level:** institutionCode: PUAC
- **Type status:** Other material. **Occurrence:** recordedBy: Aijaz A. Wachkoo; individualCount: 1; sex: worker; **Taxon:** scientificNameID: C. opaciventris; **Location:** country: India; stateProvince: Himachal Pradesh; locality: Andretta; verbatimElevation: 940 m; verbatimCoordinates: 32.0744°N, 76.5856°E; **Event:** eventDate: 12/06/2010; **Record Level:** institutionCode: PUAC
- **Type status:** Other material. **Occurrence:** recordedBy: Aijaz A. Wachkoo; individualCount: 1; sex: worker; **Taxon:** scientificNameID: C. opaciventris; **Location:** country: India; stateProvince: Himachal Pradesh; locality: Andretta; verbatimElevation: 940 m; verbatimCoordinates: 32.0744°N, 76.5856°E; **Event:** eventDate: 15/6/2010; **Record Level:** institutionCode: PUAC
- **Type status:** Other material. **Occurrence:** recordedBy: Aijaz A. Wachkoo; individualCount: 15; sex: worker; **Taxon:** scientificNameID: C. opaciventris; **Location:** country: India; stateProvince: Himachal Pradesh; locality: Andretta; verbatimElevation: 940 m; verbatimCoordinates: 32.0744°N, 76.5856°E; **Event:** eventDate: 19/6/2010; **Record Level:** institutionCode: PUAC
- **Type status:** Other material. **Occurrence:** recordedBy: Aijaz A. Wachkoo; individualCount: 3; sex: worker; **Taxon:** scientificNameID: C. opaciventris; **Location:** country: India; stateProvince: Himachal Pradesh; locality: Baijnath; verbatimElevation: 1000 m; verbatimCoordinates: 32.0527°N, 76.6500°E; **Event:** eventDate: 23/6/2010; **Record Level:** institutionCode: PUAC
- **Type status:** Other material. **Occurrence:** recordedBy: Aijaz A. Wachkoo; individualCount: 13; sex: worker; **Taxon:** scientificNameID: C. opaciventris; **Location:** country: India; stateProvince: Himachal Pradesh; locality: Bari; verbatimElevation: 520 m; verbatimCoordinates: 31.6591°N, 76.5000°E; **Event:** eventDate: 15/10/2008; **Record Level:** institutionCode: PUAC
- **Type status:** Other material. **Occurrence:** recordedBy: Aijaz A. Wachkoo; individualCount: 1; sex: worker; **Taxon:** scientificNameID: C. opaciventris; **Location:** country: India; stateProvince: Himachal Pradesh; locality: Bari; verbatimElevation: 520 m; verbatimCoordinates: 31.6591°N, 76.5000°E; **Event:** eventDate: 12/07/2010; **Record Level:** institutionCode: PUAC
- **Type status:** Other material. **Occurrence:** recordedBy: Aijaz A. Wachkoo; individualCount: 1; sex: worker; **Taxon:** scientificNameID: C. opaciventris; **Location:** country: India; stateProvince: Himachal Pradesh; locality: Dehra; verbatimElevation: 450 m; verbatimCoordinates: 31.5799°N, 76.7109°E; **Event:** eventDate: 06/07/2010; **Record Level:** institutionCode: PUAC
- **Type status:** Other material. **Occurrence:** recordedBy: Aijaz A. Wachkoo; individualCount: 1; sex: worker; **Taxon:** scientificNameID: C. opaciventris; **Location:** country: India; stateProvince: Himachal Pradesh; locality: Gagret; verbatimElevation: 600 m; verbatimCoordinates: 31.6620°N, 76.0601°E; **Event:** eventDate: 17/10/2009; **Record Level:** institutionCode: PUAC
- **Type status:** Other material. **Occurrence:** recordedBy: Aijaz A. Wachkoo; individualCount: 10; sex: worker; **Taxon:** scientificNameID: C. opaciventris; **Location:** country: India; stateProvince: Himachal Pradesh; locality: Guga; verbatimElevation: 600 m; verbatimCoordinates: 31.6864°N, 76.1898°E; **Event:** eventDate: 06/10/2008; **Record Level:** institutionCode: PUAC
- **Type status:** Other material. **Occurrence:** recordedBy: Aijaz A. Wachkoo; individualCount: 5; sex: worker; **Taxon:** scientificNameID: C. opaciventris; **Location:** country: India; stateProvince: Himachal Pradesh; locality: Guraldhar; verbatimElevation: 660 m; verbatimCoordinates: 31.6670°N, 76.4684°E; **Event:** eventDate: 16/10/2008; **Record Level:** institutionCode: PUAC
- **Type status:** Other material. **Occurrence:** recordedBy: Aijaz A. Wachkoo; individualCount: 15; sex: worker; **Taxon:** scientificNameID: C. opaciventris; **Location:** country: India; stateProvince: Himachal Pradesh; locality: Jogi Panga; verbatimElevation: 600 m; verbatimCoordinates: 31.5408°N, 76.3161°E; **Event:** eventDate: 09/10/2008; **Record Level:** institutionCode: PUAC
- **Type status:** Other material. **Occurrence:** recordedBy: Aijaz A. Wachkoo; individualCount: 3; sex: worker; **Taxon:** scientificNameID: C. opaciventris; **Location:** country: India; stateProvince: Himachal Pradesh; locality: Khatiar; verbatimElevation: 450 m; verbatimCoordinates: 32.0057°N, 75.9388°E; **Event:** eventDate: 11/10/2009; **Record Level:** institutionCode: PUAC
- **Type status:** Other material. **Occurrence:** recordedBy: Aijaz A. Wachkoo; individualCount: 1; sex: worker; **Taxon:** scientificNameID: C. opaciventris; **Location:** country: India; stateProvince: Himachal Pradesh; locality: Kotla; verbatimElevation: 500 m; verbatimCoordinates: 31.8821°N, 75.9963°E; **Event:** eventDate: 30/5/2009; **Record Level:** institutionCode: PUAC
- **Type status:** Other material. **Occurrence:** recordedBy: Aijaz A. Wachkoo; individualCount: 3; sex: worker; **Taxon:** scientificNameID: C. opaciventris; **Location:** country: India; stateProvince: Himachal Pradesh; locality: Kotla; verbatimElevation: 500 m; verbatimCoordinates: 31.8821°N, 75.9963°E; **Event:** eventDate: 29/9/2009; **Record Level:** institutionCode: PUAC
- **Type status:** Other material. **Occurrence:** recordedBy: Aijaz A. Wachkoo; individualCount: 1; sex: worker; **Taxon:** scientificNameID: C. opaciventris; **Location:** country: India; stateProvince: Himachal Pradesh; locality: Khushinagar; verbatimElevation: 760 m; verbatimCoordinates: 32.3010°N, 75.8913°E; **Event:** eventDate: 17/6/2009; **Record Level:** institutionCode: PUAC
- **Type status:** Other material. **Occurrence:** recordedBy: Aijaz A. Wachkoo; individualCount: 10; sex: worker; **Taxon:** scientificNameID: C. opaciventris; **Location:** country: India; stateProvince: Himachal Pradesh; locality: Nahan; verbatimElevation: 760 m; verbatimCoordinates: 30.5596°N, 77.2960°E; **Event:** eventDate: 04/06/2010; **Record Level:** institutionCode: PUAC
- **Type status:** Other material. **Occurrence:** recordedBy: Aijaz A. Wachkoo; individualCount: 1; sex: worker; **Taxon:** scientificNameID: C. opaciventris; **Location:** country: India; stateProvince: Himachal Pradesh; locality: Pong Dam; verbatimElevation: 450 m; verbatimCoordinates: 31.9710°N, 75.9469°E; **Event:** eventDate: 18/10/2009; **Record Level:** institutionCode: PUAC
- **Type status:** Other material. **Occurrence:** recordedBy: Aijaz A. Wachkoo; individualCount: 1; sex: worker; **Taxon:** scientificNameID: C. opaciventris; **Location:** country: India; stateProvince: Himachal Pradesh; locality: Siholi; verbatimElevation: 560 m; verbatimCoordinates: 31.9456°N, 75.9949°E; **Event:** eventDate: 08/07/2010; **Record Level:** institutionCode: PUAC
- **Type status:** Other material. **Occurrence:** recordedBy: Aijaz A. Wachkoo; individualCount: 1; sex: worker; **Taxon:** scientificNameID: C. opaciventris; **Location:** country: India; stateProvince: Himachal Pradesh; locality: Siholi; verbatimElevation: 560 m; verbatimCoordinates: 31.9456°N, 75.9949°E; **Event:** eventDate: 08/07/2010; **Record Level:** institutionCode: PUAC
- **Type status:** Other material. **Occurrence:** recordedBy: Aijaz A. Wachkoo; individualCount: 1; sex: worker; **Taxon:** scientificNameID: C. opaciventris; **Location:** country: India; stateProvince: Himachal Pradesh; locality: Siholi; verbatimElevation: 560 m; verbatimCoordinates: 31.9456°N, 75.9949°E; **Event:** eventDate: 08/07/2010; **Record Level:** institutionCode: PUAC
- **Type status:** Other material. **Occurrence:** recordedBy: Aijaz A. Wachkoo; individualCount: 5; sex: worker; **Taxon:** scientificNameID: C. opaciventris; **Location:** country: India; stateProvince: Himachal Pradesh; locality: Terrace; verbatimElevation: 420 m; verbatimCoordinates: 31.9285°N, 75.9313°E; **Event:** eventDate: 12/10/2009; **Record Level:** institutionCode: PUAC
- **Type status:** Other material. **Occurrence:** recordedBy: Aijaz A. Wachkoo; individualCount: 1; sex: worker; **Taxon:** scientificNameID: C. opaciventris; **Location:** country: India; stateProvince: Himachal Pradesh; locality: Terrace; verbatimElevation: 420 m; verbatimCoordinates: 31.9285°N, 75.9313°E; **Event:** eventDate: 15/7/2010; **Record Level:** institutionCode: PUAC
- **Type status:** Other material. **Occurrence:** recordedBy: Aijaz A. Wachkoo; individualCount: 1; sex: queen; **Taxon:** scientificNameID: C. opaciventris; **Location:** country: India; stateProvince: Himachal Pradesh; locality: Una; verbatimElevation: 400 m; verbatimCoordinates: 31.4685°N, 76.2709°E; **Event:** eventDate: 05/10/2008; **Record Level:** institutionCode: PUAC
- **Type status:** Other material. **Occurrence:** recordedBy: Aijaz A. Wachkoo; individualCount: 2; sex: worker; **Taxon:** scientificNameID: C. opaciventris; **Location:** country: India; stateProvince: Jammu and Kashmir; locality: Mansar; verbatimElevation: 690 m; verbatimCoordinates: 32.6979°N, 75.1489°E; **Event:** eventDate: 03/08/2010; **Record Level:** institutionCode: PUAC
- **Type status:** Other material. **Occurrence:** recordedBy: Aijaz A. Wachkoo; individualCount: 1; sex: worker; **Taxon:** scientificNameID: C. opaciventris; **Location:** country: India; stateProvince: Jammu and Kashmir; locality: Sukrala; verbatimElevation: 1040 m; verbatimCoordinates: 32.6528°N, 75.5817°E; **Event:** eventDate: 07/08/2010; **Record Level:** institutionCode: PUAC
- **Type status:** Other material. **Occurrence:** recordedBy: Aijaz A. Wachkoo; individualCount: 7; sex: worker; **Taxon:** scientificNameID: C. opaciventris; **Location:** country: India; stateProvince: Jammu and Kashmir; locality: Udhampur; verbatimElevation: 690 m; verbatimCoordinates: 32.9141°N, 75.1424°E; **Event:** eventDate: 04/07/2009; **Record Level:** institutionCode: PUAC
- **Type status:** Other material. **Occurrence:** recordedBy: Aijaz A. Wachkoo; individualCount: 1; sex: queen; **Taxon:** scientificNameID: C. opaciventris; **Location:** country: India; stateProvince: Punjab; locality: Dunera; verbatimElevation: 700 m; verbatimCoordinates: 32.4443°N, 75.8900°E; **Event:** eventDate: 23/6/2009; **Record Level:** institutionCode: PUAC
- **Type status:** Other material. **Occurrence:** recordedBy: Aijaz A. Wachkoo; individualCount: 2; sex: male; **Taxon:** scientificNameID: C. opaciventris; **Location:** country: India; stateProvince: Punjab; locality: Dunera; verbatimElevation: 700 m; verbatimCoordinates: 32.4443°N, 75.8900°E; **Event:** eventDate: 24/6/2009; **Record Level:** institutionCode: PUAC
- **Type status:** Other material. **Occurrence:** recordedBy: Aijaz A. Wachkoo; individualCount: 12; sex: worker; **Taxon:** scientificNameID: C. opaciventris; **Location:** country: India; stateProvince: Uttarakhand; locality: Assan Barrage; verbatimElevation: 750 m; verbatimCoordinates: 30.4417°N, 77.6754°E; **Event:** eventDate: 10/05/2009; **Record Level:** institutionCode: PUAC
- **Type status:** Other material. **Occurrence:** recordedBy: Aijaz A. Wachkoo; individualCount: 1; sex: worker; **Taxon:** scientificNameID: C. opaciventris; **Location:** country: India; stateProvince: Uttarakhand; locality: Assan Barrage; verbatimElevation: 750 m; verbatimCoordinates: 30.4417°N, 77.6754°E; **Event:** eventDate: 21/8/2009; **Record Level:** institutionCode: PUAC
- **Type status:** Other material. **Occurrence:** recordedBy: Shahid A. Akbar; individualCount: 6; sex: worker; **Taxon:** scientificNameID: *C. sericeus*s; **Location:** country: India; stateProvince: Karnataka; locality: Gundlupet; verbatimElevation: 800 m; verbatimCoordinates: 11.8142°N, 76.6991°E; **Event:** eventDate: 12/10/2010; **Record Level:** institutionCode: PUAC
- **Type status:** Other material. **Occurrence:** recordedBy: Shahid A. Akbar; individualCount: 1; sex: queen; **Taxon:** scientificNameID: C. sericeus; **Location:** country: India; stateProvince: Karnataka; locality: Gundlupet; verbatimElevation: 800 m; verbatimCoordinates: 11.8142°N, 76.6991°E; **Event:** eventDate: 12/10/2010; **Record Level:** institutionCode: PUAC
- **Type status:** Other material. **Occurrence:** recordedBy: Shahid A. Akbar; individualCount: 2; sex: worker; **Taxon:** scientificNameID: C. sericeus; **Location:** country: India; stateProvince: Karnataka; locality: Gundlupet; verbatimElevation: 800 m; verbatimCoordinates: 11.8142°N, 76.6991°E; **Event:** eventDate: 13/10/2010; **Record Level:** institutionCode: PUAC
- **Type status:** Other material. **Occurrence:** recordedBy: Shahid A. Akbar; individualCount: 4; sex: worker; **Taxon:** scientificNameID: C. sericeus; **Location:** country: India; stateProvince: Karnataka; locality: Mangalore; verbatimElevation: 50 m; verbatimCoordinates: 12.9161°N, 74.8635°E; **Event:** eventDate: 22/10/2010; **Record Level:** institutionCode: PUAC
- **Type status:** Other material. **Occurrence:** recordedBy: Shahid A. Akbar; individualCount: 3; sex: worker; **Taxon:** scientificNameID: C. sericeus; **Location:** country: India; stateProvince: Kerala; locality: Mukkali; verbatimElevation: 658 m; verbatimCoordinates: 11.0602°N, 76.5395°E; **Event:** eventDate: 29/09/2011; **Record Level:** institutionCode: PUAC
- **Type status:** Other material. **Occurrence:** recordedBy: Shahid A. Akbar; individualCount: 1; sex: queen; **Taxon:** scientificNameID: C. sericeus; **Location:** country: India; stateProvince: Kerala; locality: Mukkali; verbatimElevation: 658 m; verbatimCoordinates: 11.0602°N, 76.5395°E; **Event:** eventDate: 29/09/2011; **Record Level:** institutionCode: PUAC
- **Type status:** Other material. **Occurrence:** recordedBy: Shahid A. Akbar; individualCount: 3; sex: worker; **Taxon:** scientificNameID: C. sericeus; **Location:** country: India; stateProvince: Kerala; locality: Periyar Tiger Reserve; verbatimElevation: 1005 m; verbatimCoordinates: 9.4688°N, 77.2109°E; **Event:** eventDate: 05/10/2011; **Record Level:** institutionCode: PUAC
- **Type status:** Other material. **Occurrence:** recordedBy: Shahid A. Akbar; individualCount: 3; sex: worker; **Taxon:** scientificNameID: C. sericeus; **Location:** country: India; stateProvince: Kerala; locality: Shreekaryam; verbatimElevation: 50 m; verbatimCoordinates: 8.5464°N, 76.9142°E; **Event:** eventDate: 15/09/2010; **Record Level:** institutionCode: PUAC
- **Type status:** Other material. **Occurrence:** recordedBy: Shahid A. Akbar; individualCount: 5; sex: worker; **Taxon:** scientificNameID: C. sericeus; **Location:** country: India; stateProvince: Kerala; locality: Shreekaryam; verbatimElevation: 50 m; verbatimCoordinates: 8.5464°N, 76.9142°E; **Event:** eventDate: 15/09/2010; **Record Level:** institutionCode: PUAC
- **Type status:** Other material. **Occurrence:** recordedBy: Shahid A. Akbar; individualCount: 2; sex: worker; **Taxon:** scientificNameID: C. sericeus; **Location:** country: India; stateProvince: Kerala; locality: Silent Valley National Park; verbatimElevation: 897 m; verbatimCoordinates: 11.0682°N, 76.5191°E; **Event:** eventDate: 20/09/2011; **Record Level:** institutionCode: PUAC
- **Type status:** Other material. **Occurrence:** recordedBy: Shahid A. Akbar; individualCount: 1; sex: queen; **Taxon:** scientificNameID: C. sericeus; **Location:** country: India; stateProvince: Kerala; locality: Silent Valley National Park; verbatimElevation: 897 m; verbatimCoordinates: 11.0682°N, 76.5191°E; **Event:** eventDate: 20/09/2011; **Record Level:** institutionCode: PUAC
- **Type status:** Other material. **Occurrence:** recordedBy: Shahid A. Akbar; individualCount: 2; sex: worker; **Taxon:** scientificNameID: C. sericeus; **Location:** country: India; stateProvince: Kerala; locality: Silent Valley National Park; verbatimElevation: 897 m; verbatimCoordinates: 11.0682°N, 76.5191°E; **Event:** eventDate: 24/09/2011; **Record Level:** institutionCode: PUAC
- **Type status:** Other material. **Occurrence:** recordedBy: Shahid A. Akbar; individualCount: 5; sex: worker; **Taxon:** scientificNameID: C. sericeus; **Location:** country: India; stateProvince: Kerala; locality: Silent Valley National Park; verbatimElevation: 897 m; verbatimCoordinates: 11.0682°N, 76.5191°E; **Event:** eventDate: 25/09/2011; **Record Level:** institutionCode: PUAC
- **Type status:** Other material. **Occurrence:** recordedBy: Shahid A. Akbar; individualCount: 4; sex: worker; **Taxon:** scientificNameID: C. sericeus; **Location:** country: India; stateProvince: Kerala; locality: Silent Valley National Park; verbatimElevation: 897 m; verbatimCoordinates: 11.0682°N, 76.5191°E; **Event:** eventDate: 26/09/2011; **Record Level:** institutionCode: PUAC
- **Type status:** Other material. **Occurrence:** recordedBy: Shahid A. Akbar; individualCount: 3; sex: worker; **Taxon:** scientificNameID: C. sericeus; **Location:** country: India; stateProvince: Kerala; locality: University of Calicut; verbatimElevation: 2 m; verbatimCoordinates: 11.1345°N, 75.8951°E; **Event:** eventDate: 23/10/2013; **Record Level:** institutionCode: PUAC
- **Type status:** Other material. **Occurrence:** recordedBy: Shahid A. Akbar; individualCount: 3; sex: worker; **Taxon:** scientificNameID: C. sericeus; **Location:** country: India; stateProvince: Tamil Nadu; locality: Kanyakumari; verbatimElevation: 20 m; verbatimCoordinates: 8.0878°N, 77.5392°E; **Event:** eventDate: 24/09/2010; **Record Level:** institutionCode: PUAC

#### Description

**Worker** Fig. 1

Morphometric data: HL 1.52-2.58; HW 1.44-2.85; EL 0.45-0.66; SL 1.45-1.94; PnW 1.30-2.14; ML 2.10-3.16; MTL 1.32-1.94; HTL 1.86-2.79; PL 0.40-0.57; GL 2.02-3.69; TL 6.04-10.00 mm. Indices: CI 95-110; SI 68-101; REL 26-30 (n=25).

Head triangular, wider than long in major worker, lateral margins arched, posterior margin transverse to gently convex; scapes short, surpassing posterior margin of head by less than one-fifth their length; head of minor worker subquadrate longer than wide, with gently arched lateral margins and convex posterior margin; scapes of minor worker longer, surpassing posterior margin of head by one-fourth their length; clypeus subcarinate; anterior clypeal margin emarginate in major worker, convex in minor worker not projected beyond anterior margin of gena; anterolateral corners broadly rounded; mandibles subtriangular, robust, armed with 5-teeth.

Mesosomal outline in profile interrupted at deep metanotal groove; promesonotum forming a regular convexity, metanotum forming an angle with mesonotum; basal portion of propodeum horizontal or slightly concave; apical portion excavate; propodeum forming acute angle with declivous face; propodeal spiracle elongate, slit-like; petiole nodiform, in profile, uniformly wide anteroposteriorly, dorsally rounded; hind tibiae tubular.

Body opaque: head, mesonotum and gaster microreticulate, reticulations feebler on mesopleuron, petiole, scapes and legs; mandibles punctured.

Body covered sparsely with short appressed pubescence, denser on gaster but not hiding the cuticular sculpture; whole body covered with erect setae; setae on propodeum denser than promesonotum; hindtibia with irregular multiple rows of spiny bristles in addition to 3-4 suberect setae at distal end near spurs.

Head and antennae reddish, remainder of body dull black.

**Queen** Fig. 2a, b, c

Morphometric data: HL 2.34-2.46; HW 2.52-2.55; EL 0.69-0.75; SL 1.88-1.90; ML 3.96-3.97; MTL 1.86-1.92; HTL 2.69-2.73; PL 0.60-0.63; GL 5.27-5.39; TL 12.17-12.45 mm. Indices: CI 104-108; SI 75-80; REL 29-30 (n=2).

As in major worker, with modifications expected for caste and the following differences: head squarer than in major worker; mesosoma and gaster densely pubescent; mandibles 5-toothed; scutum and scutellum with shallow widely spaced punctures; declivous face right angle.

**Male** Fig. 2d, e, f

Morphometric data: HL 1.30-1.34; HW 1.30-1.33; EL 0.56-0.57; SL 1.44-1.47, ML 3.48-3.49; MTL 1.76-1.78; HTL 2.21-2.26; PL 0.43-0.45; GL 3.40-3.49; TL 8.61-8.77 mm. Indices: CI 99-100; SI 108-113; REL 42-43 (n=2).

Head wider than long including eyes; scapes long, surpassing posterior margin of head by about two-fifths their length; clypeus subcarinate in middle with nearly transverse anterolateral corners; mandibles slender, curved strap like, apical tooth acute, remainder without any teeth or denticles; when closed their tips overlap.

In anterior view, petiolar dorsal margin with two subtle apices; propodeal declivity smoothly rounded; propodeal spiracle elongate, slit shaped.

Pygostyles distally setose; basimeres large, broad at the base with bluntly rounded apex; in dorsal view, telomeres elongate anteroposteriorly and curved inward; in lateral view tubular, rounded apically covered by punctures; basimeres with sparse setae, telomeres abundantly setose; cuspides small bent toward digiti; digiti much longer than cuspides, about 2 times the length of cuspides bent towards each other apically; penisvalva projecting with apices of each penisvalva directed posterolaterally.

Vestiture as in major worker with following differences: propodeum shiny without any setae on dorsum; gaster less pubescent than in other conspecific castes.

Colour dull black; gena, mandibles and antennae brownish.

#### Ecology

These ants have been observed to form nests in sandy soils, dry soils and under large stones. The minor workers were collected largely individually at foraging while most of the major workers remained inside the nests.

#### Taxon discussion

*Camponotus opaciventris* most resembles to and is often confused with *C. sericeus* (Fig. 3) from which it is distinguished by the reddish head (vs. blackish) and gaster without a dense layer of pubescence obscuring cuticular sculpture (vs. dense pubescence layer present and obscuring the cuticular sculpture underneath). *Camponotus opaciventris* seems to be general in distribution in the Western Himalayas (mostly restricted upto the altitudes of 1200 m). However, *C. opaciventris* has also been reported from other parts of India occurring sympatrically with *C. sericeus* which eludes the possibility that *C. opaciventris* is simply differentiated due to allopatric reduction of gene flow (Forel 1892, Narendra 2006, Akbar 2014). The two species seem to retain their distinctiveness whenever they occur in sympatry. On the other hand *C. sericeus* is widespread in most of the India but does not seem to extend its distribution in the Western Himalayas. On reexamining the material of earlier records published from Western Himalayas it becomes apparent that *C. opaciventris* has been misidentified as *C. sericeus*. The character differences observed for both the species are consistent throughout the range and there is definitely no overlap of characters and no indication for intraspecific variation.

## Supplementary Material

XML Treatment for Camponotus opaciventris

## Figures and Tables

**Figure 1a.**
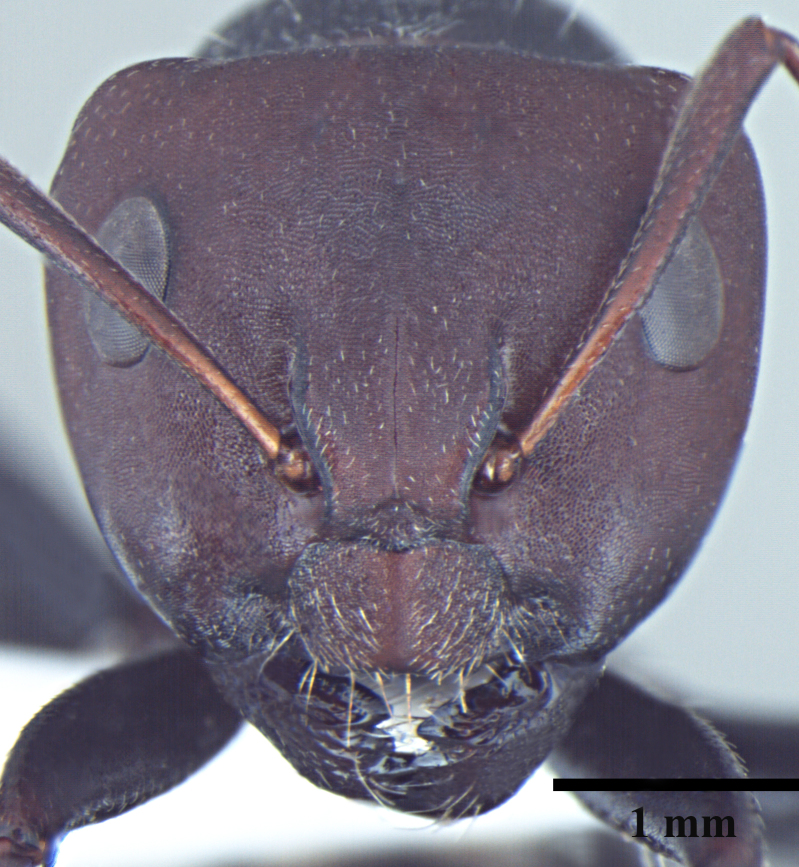
Head, full-face view (major worker)

**Figure 1b.**
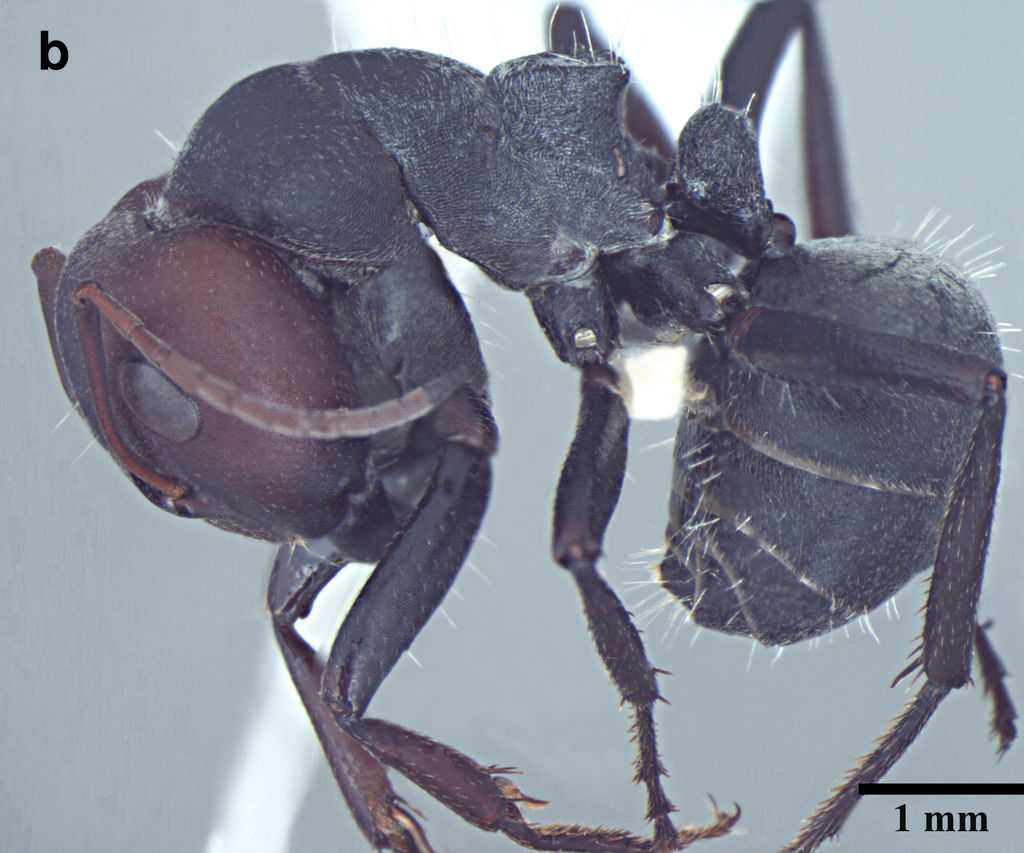
Body, lateral view (major worker)

**Figure 1c.**
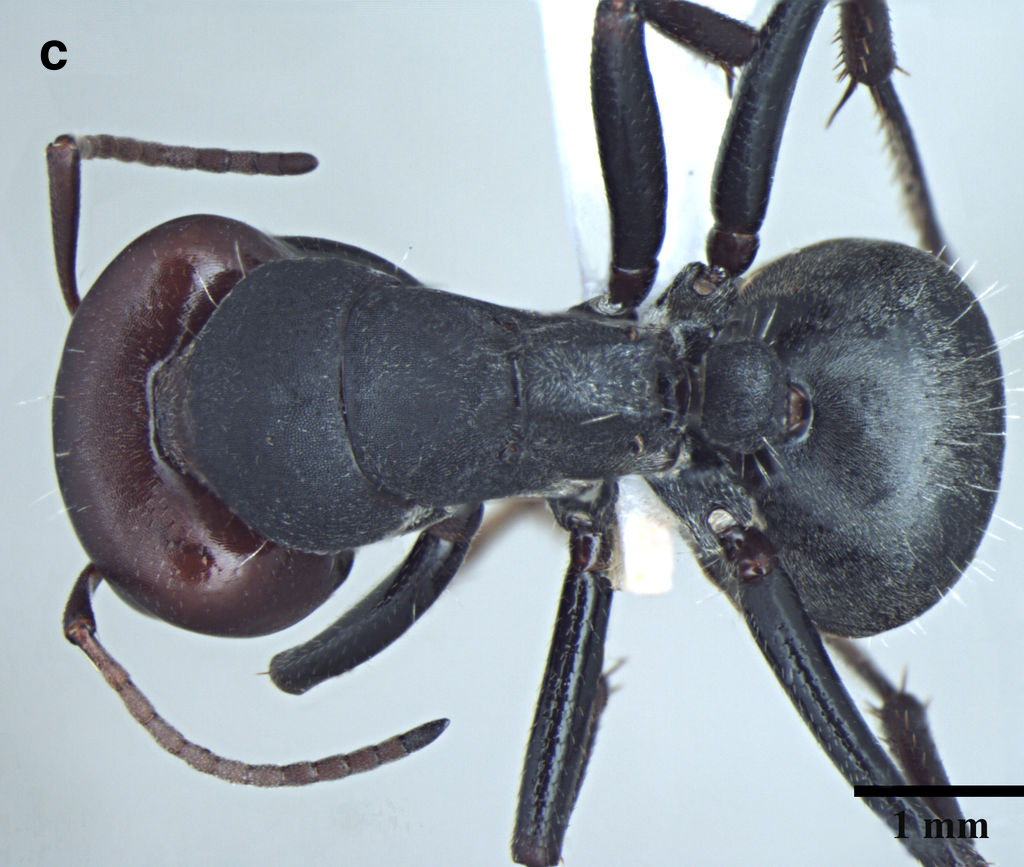
Body, dorsal view (major worker)

**Figure 1d.**
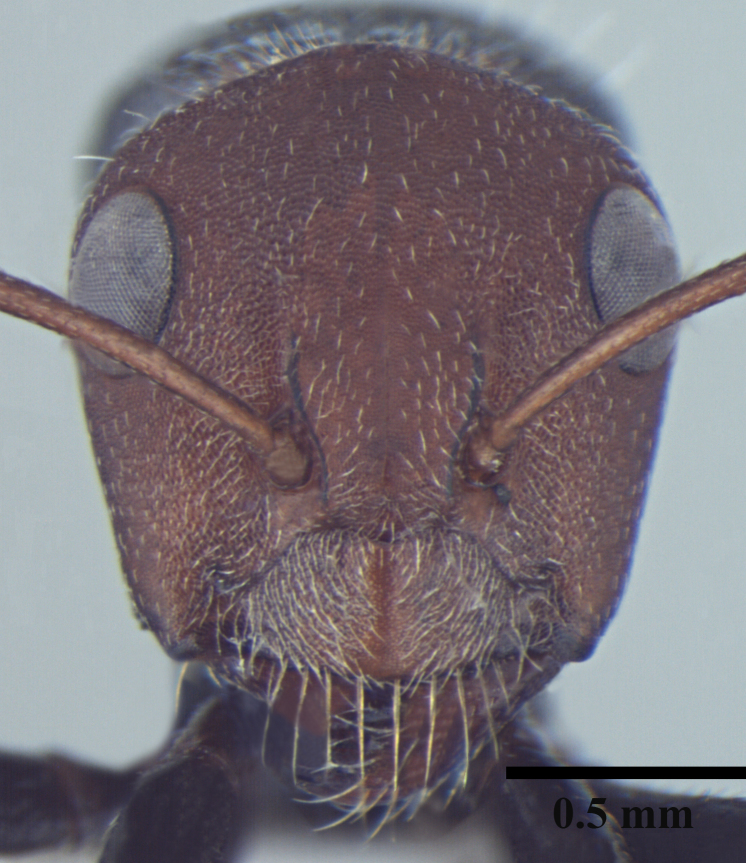
Head, full-face view (minor worker)

**Figure 1e.**
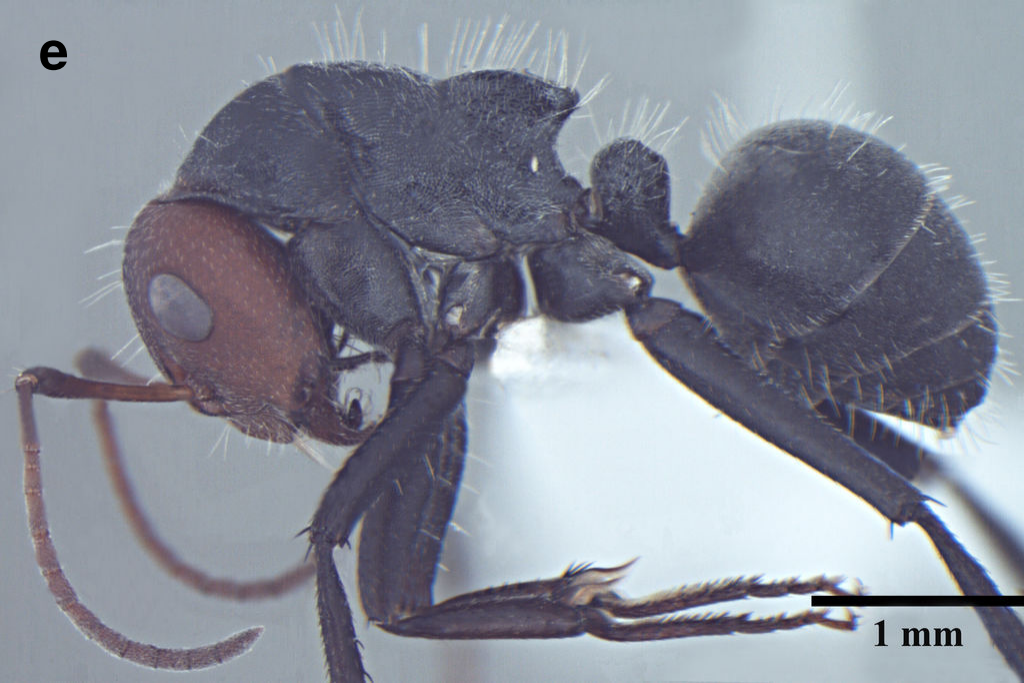
Body, lateral view (minor worker)

**Figure 1f.**
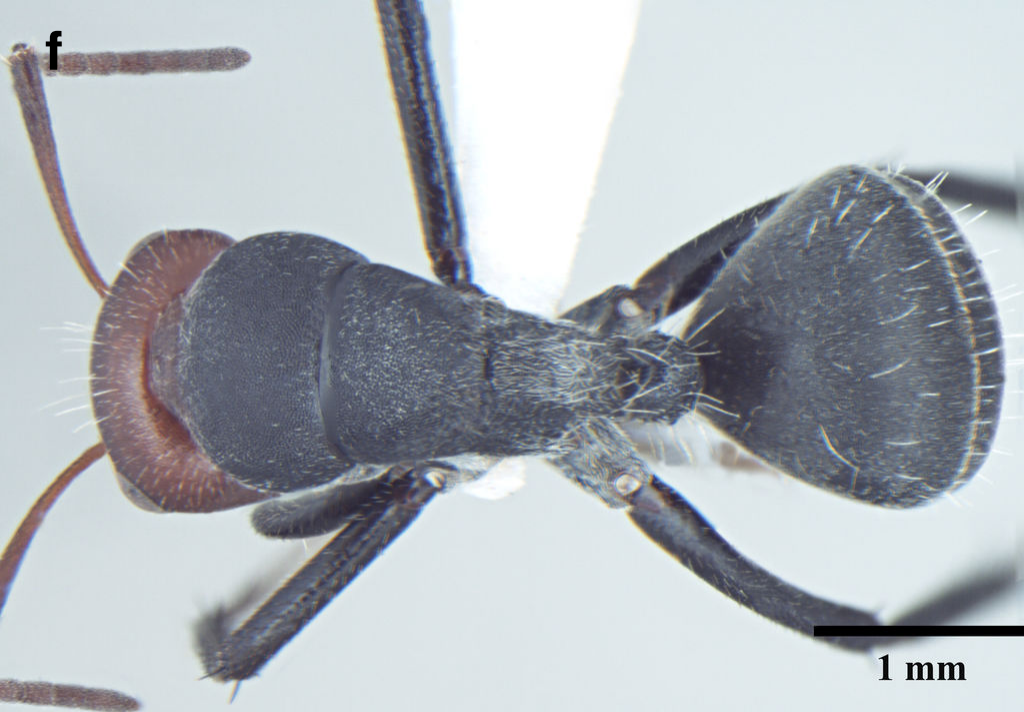
Body, dorsal view (minor worker)

**Figure 2a.**
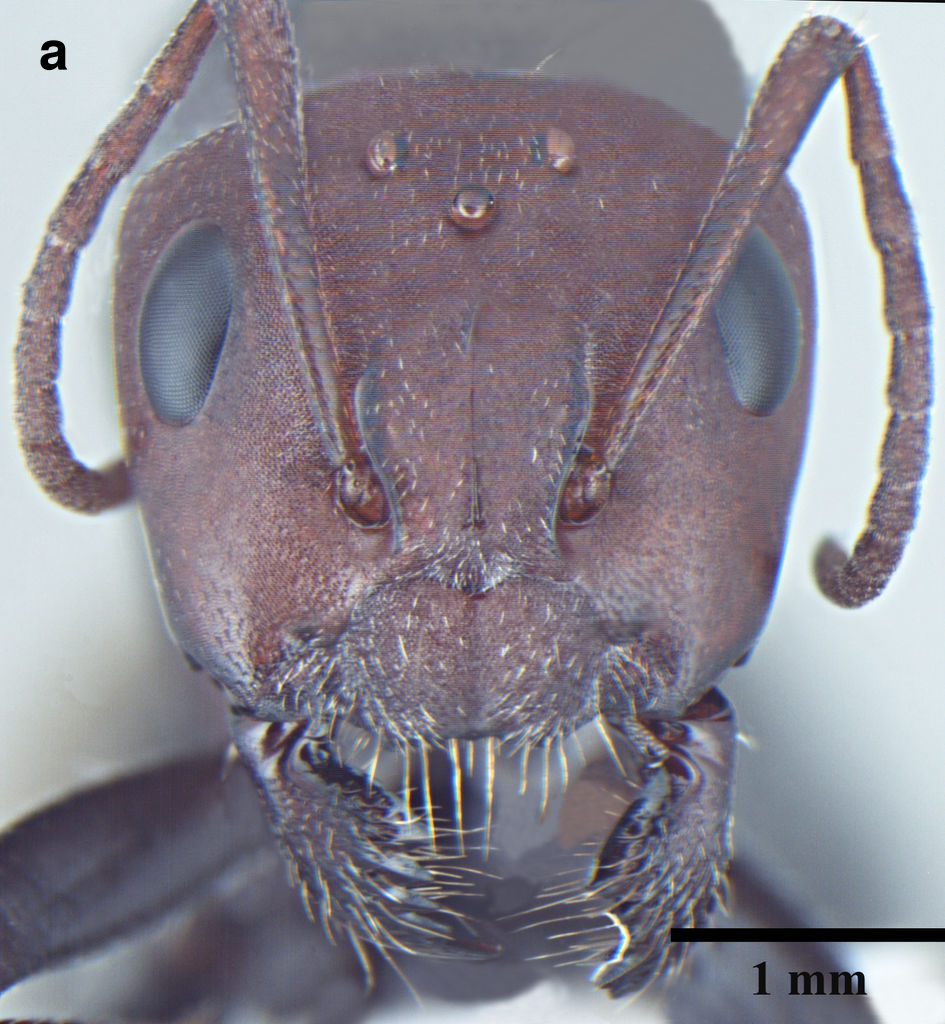
Head, full-face view (queen)

**Figure 2b.**
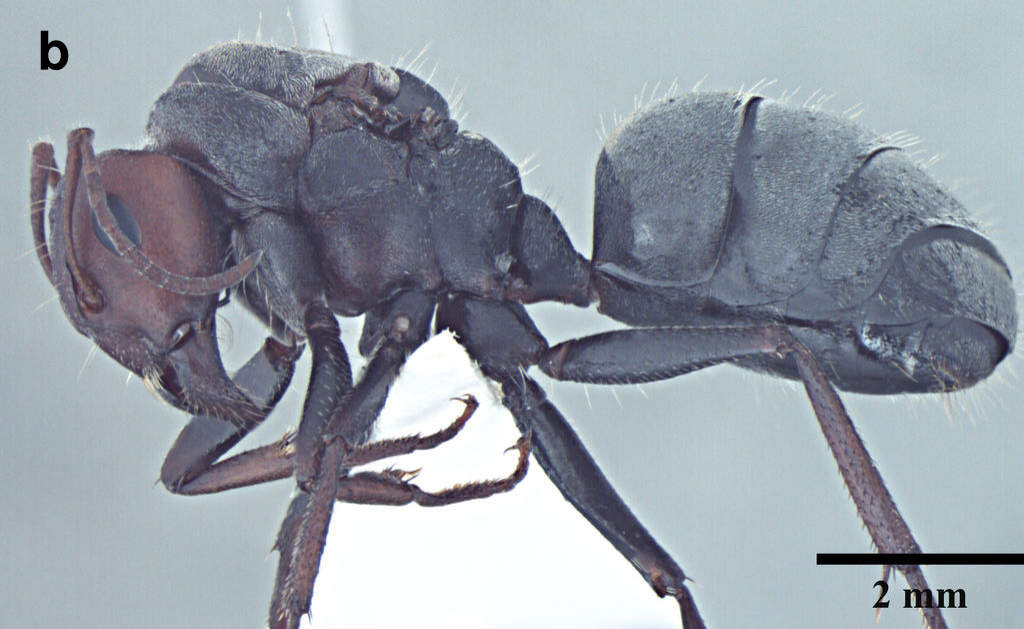
Body, lateral view (queen)

**Figure 2c.**
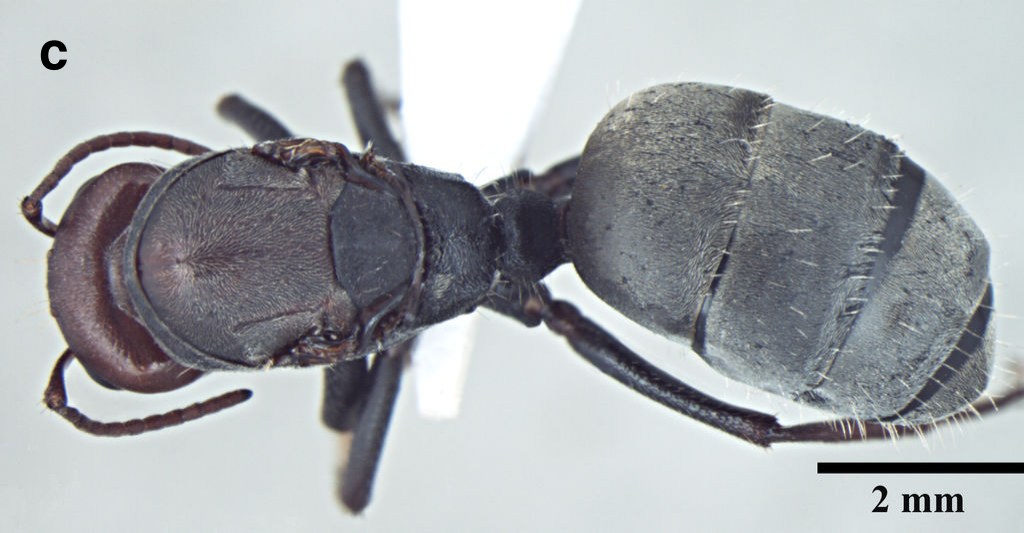
Body, dorsal view (queen)

**Figure 2d.**
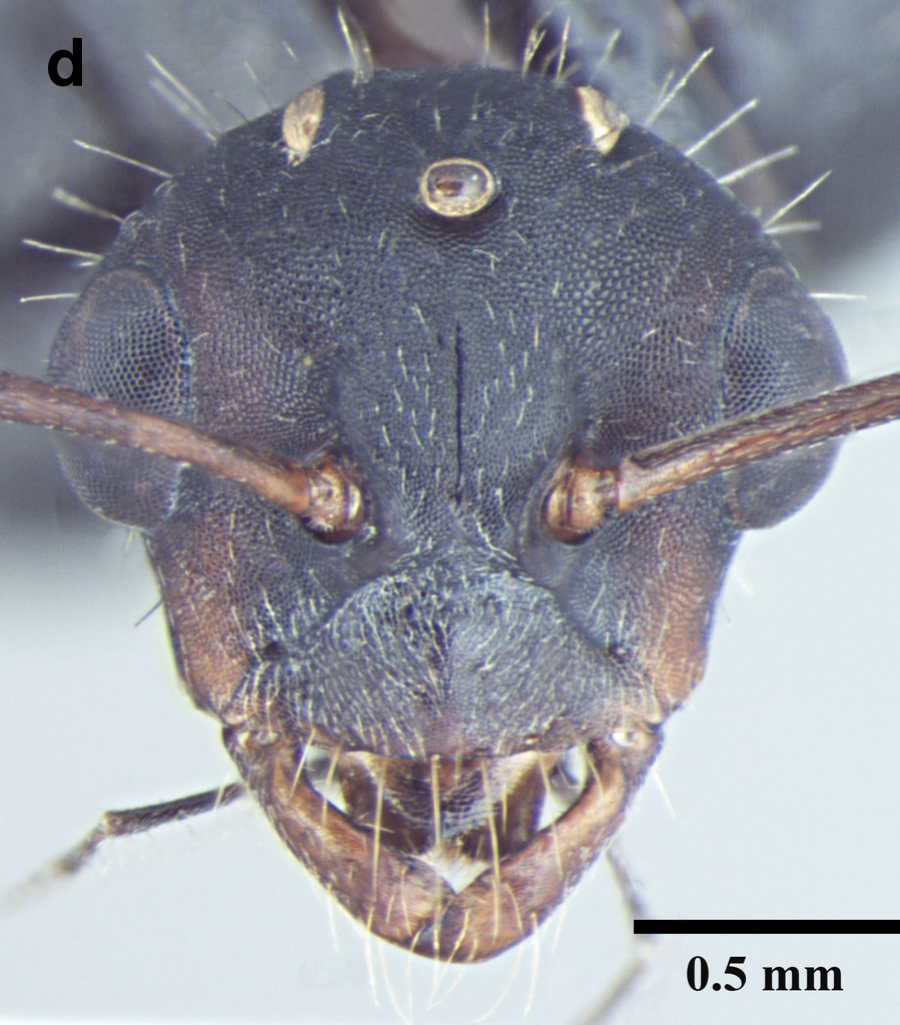
Head, full-face view (male)

**Figure 2e.**
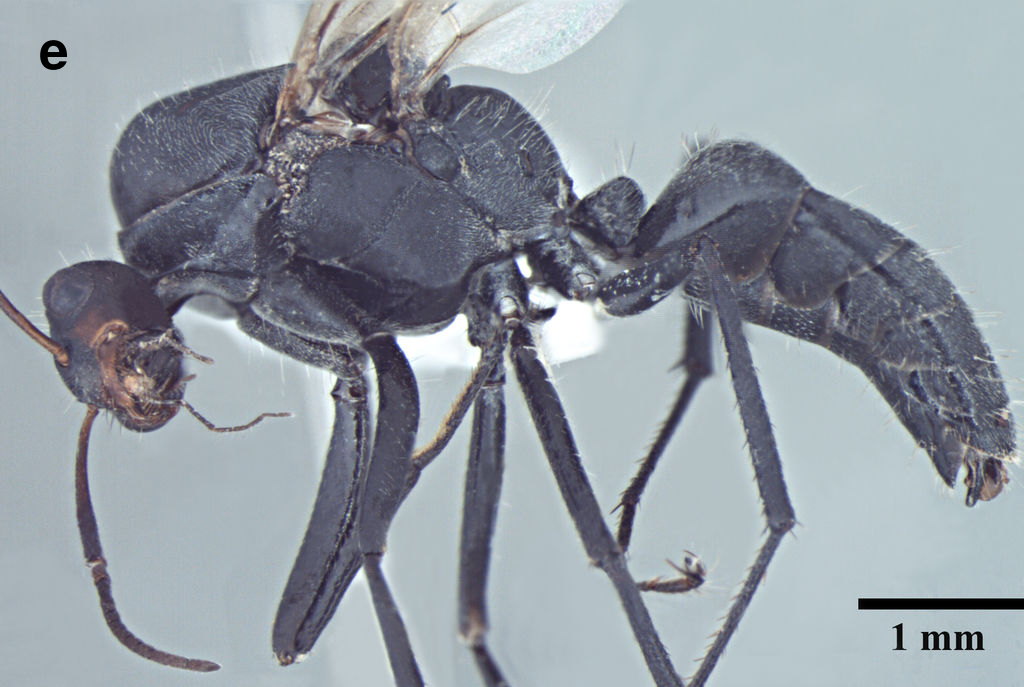
Body, lateral view (male)

**Figure 2f.**
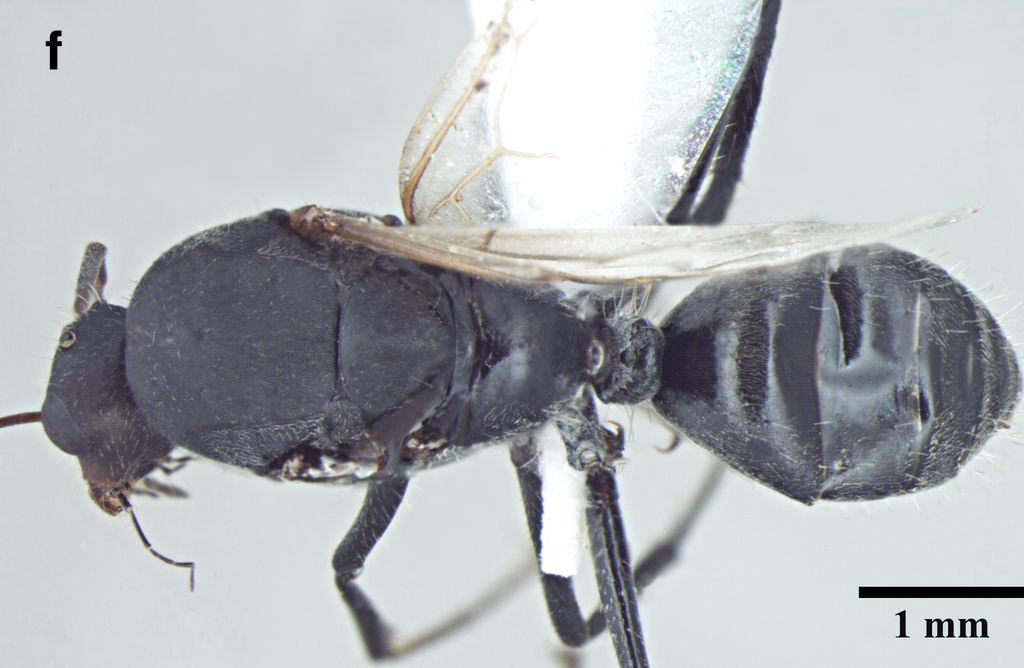
Body, dorsal view (male)

**Figure 3a.**
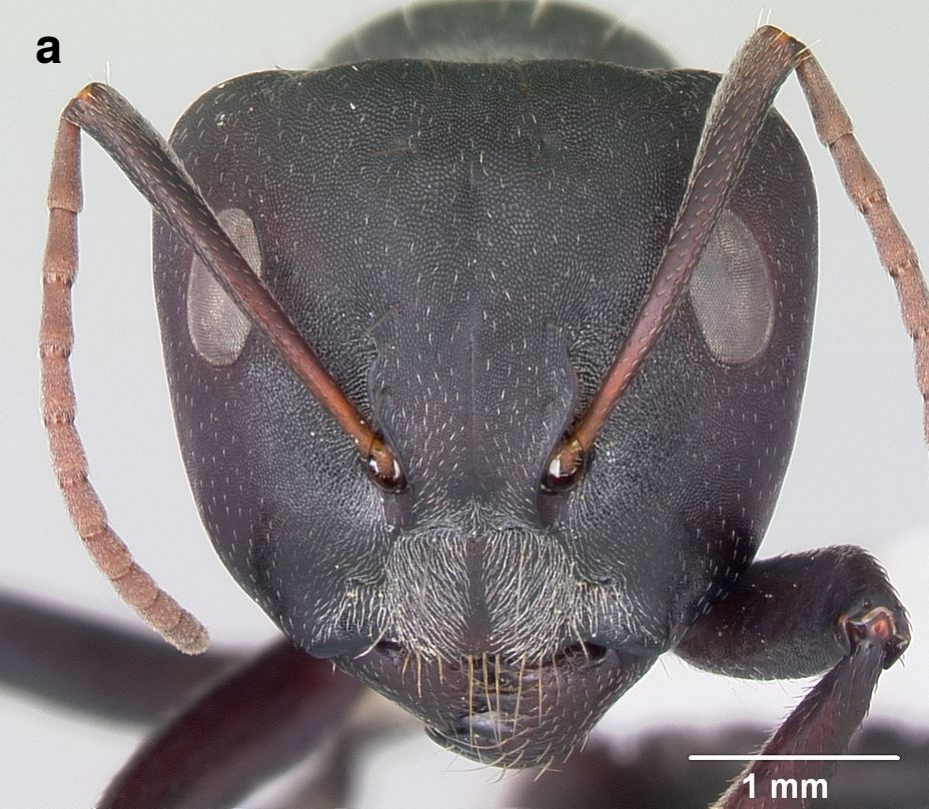
Head, full-face view (major worker); AntWeb: CASENT0104896, courtesy April Nobile

**Figure 3b.**
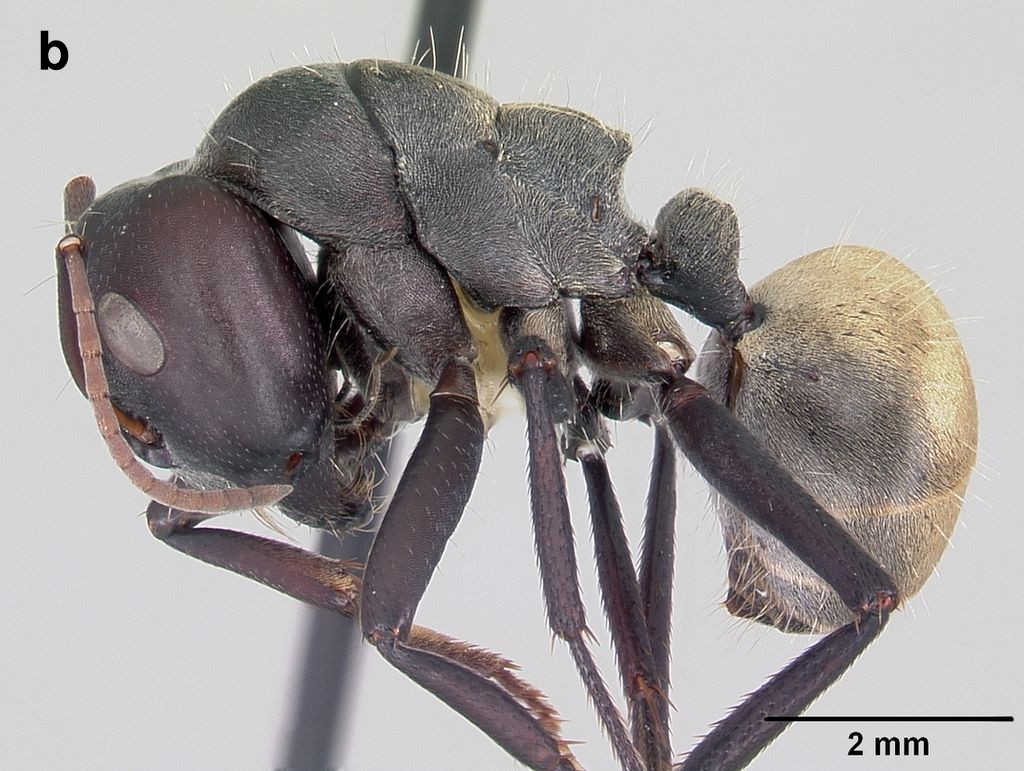
Body, lateral view (major worker); AntWeb: CASENT0104896, courtesy April Nobile

**Figure 3c.**
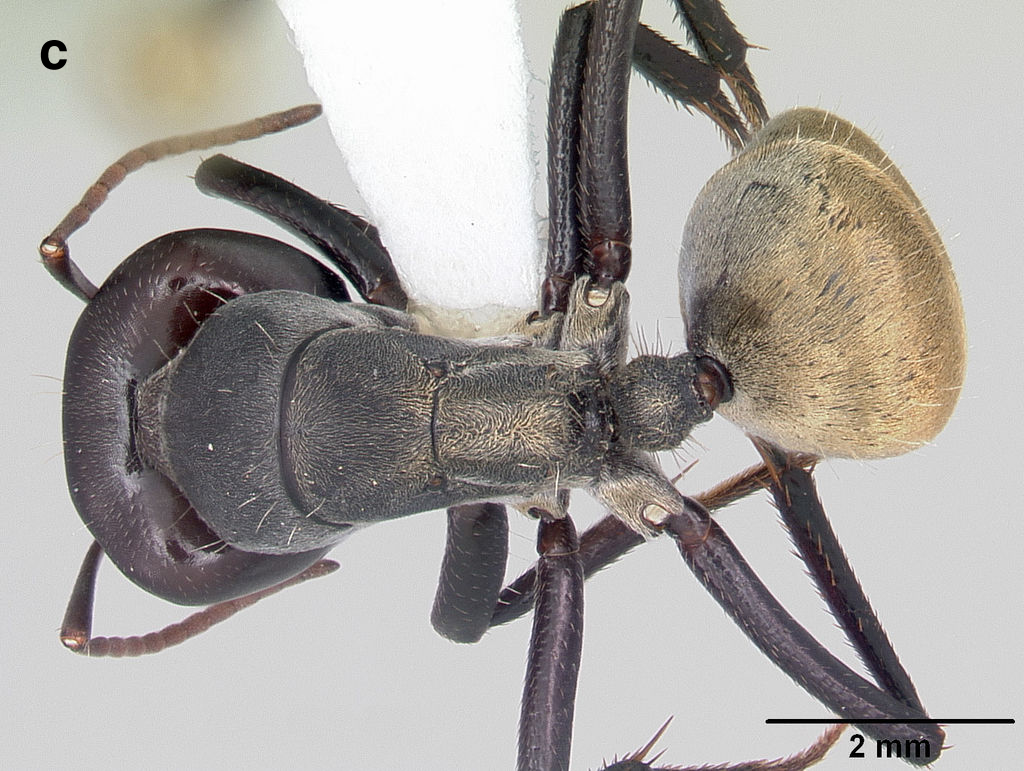
Body, dorsal view (major worker); AntWeb: CASENT0104896, courtesy April Nobile

**Figure 3d.**
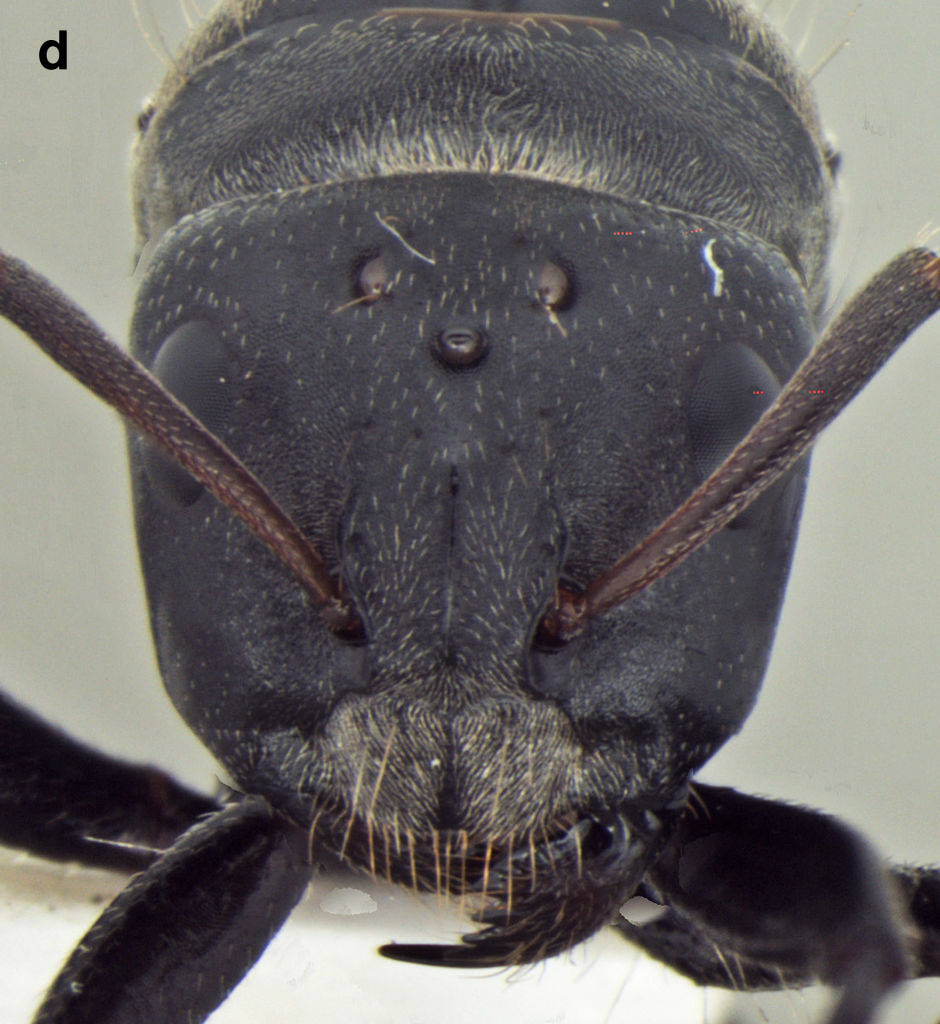
Head, full-face view (queen)

**Figure 3e.**
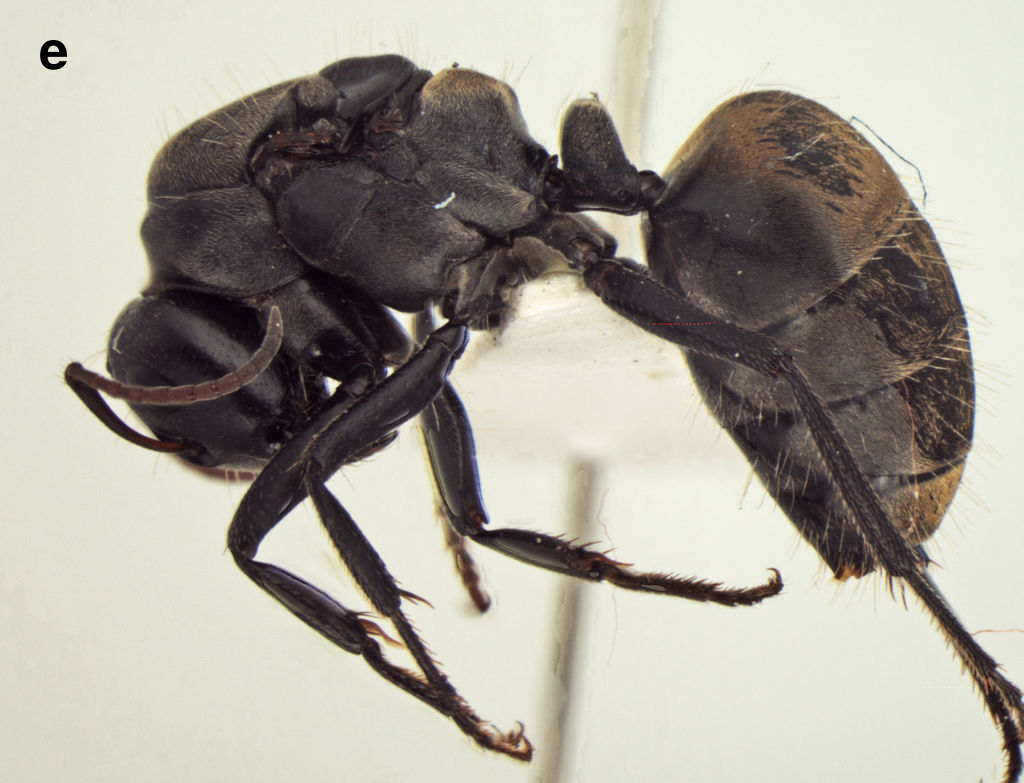
Body, lateral view (queen); cuticle obscuring pubescence of gaster abraded in specimen

**Figure 3f.**
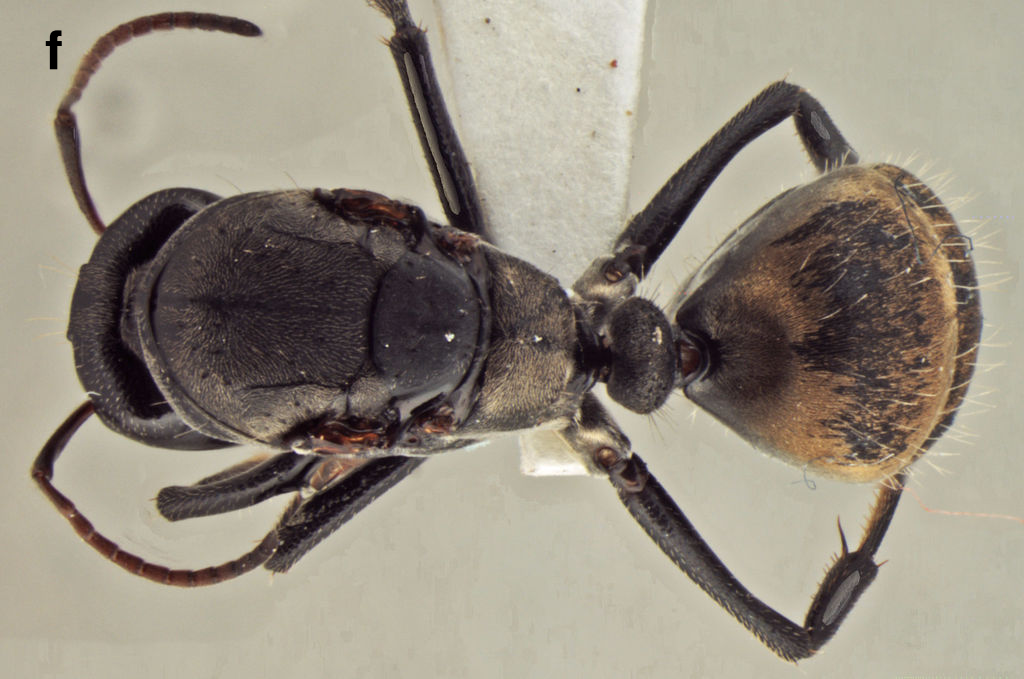
Body, dorsal view (queen); cuticle obscuring pubescence of gaster abraded in specimen

## References

[B3426994] Akbar S. A. (2014). Taxonomic Studies of Ants (Formicidae:Hymenoptera) from Western Ghats of India.

[B3398028] AntWeb Bolton World Catalog. https://www.antweb.org/description.do?subfamily=formicinae&genus=camponotus&rank=genus&project=worldants.

[B3397979] Bharti H., Wachkoo A. A. (2014). A new carpenter ant, *Camponotus
parabarbatus* (Hymenoptera: Formicidae) from India. Biodiversity Data Journal.

[B3397958] Bharti H., Wachkoo A. A. (2015). Neotype designation and redescription of *Camponotus
horseshoetus* (Hymenoptera: Formicidae). Acta Entomologica Musei Nationalis Pragae.

[B3397999] Bharti H., Guénard B., Bharti M., Economo E. P. (2016). An updated checklist of the ants of India with their specific distributions in Indian states (Hymenoptera, Formicidae). ZooKeys.

[B3398019] Bingham C. T. (1903). The fauna of British India, including Ceylon and Burma. Hymenoptera, Vol. II. Ants and Cuckoo-wasps.

[B3398037] Boudinot B. (2013). The male genitalia of ants: musculature, homology, and functional morphology (Hymenoptera, Aculeata, Formicidae). Journal of Hymenoptera Research.

[B3398047] Dietrich C. O. (2004). Taxonomische Beiträge zur Myrmekofauna Jordaniens (Hymenoptera: Formicidae). Denisia.

[B3398060] Emery C. (1896). Saggio di un catalogo sistematico dei generi *Camponotus*, *Polyrhachis* e affini. Memorie della Reale Accademia delle Scienze dell'Istituto di Bologna.

[B3398070] Emery C. (1925). Hymenoptera. Fam. Formicidae. Subfam. Formicinae. Genera Insectorum.

[B3398080] Fabricius J. C. (1798). Supplementum entomologiae systematicae.

[B3398090] Forel A, (1892). Les Formicides de l'Empire des Indes et de Ceylan. Part I. Journal of the Bombay Natural History Society.

[B3398100] Forel A, (1908). Fourmis de Ceylan et d'Égypte récoltées par le Prof. E. Bugnion. Lasius carniolicus. Fourmis de Kerguelen. Pseudandrie? Strongylognathus testaceus. Bulletin de la Société Vaudoise des Sciences Naturelles.

[B3398134] Hölldobler B., Wilson E. O. (1990). The Ants.

[B3398159] Mayr G. (1861). Die Europäischen Formiciden.

[B3398176] Mayr G. (1879). Beiträge zur Ameisen-Fauna Asiens. Verhandlungen der Kaiserlich-Königlichen. Zoologisch-Botanischen Gesellschaft in Wien.

[B3426985] Narendra A. Ant Visions. https://antvisions.wordpress.com/2006/11/22/rare-morph-of-camponotus-Camponotussericeus-bangalore/.

[B3398187] Radchenko A. G. (1996). A key to the ant genus *Camponotus* (Hymenoptera, Formicidae) in Palearctic Asia. Zoologicheskii Zhurnal.

[B3398197] Shah G. M., Jan U., Wachkoo A. A. (2014). A checklist of Hoverflies (Diptera: Syrphidae) in the Western Himalaya, India. Acta Zoologica Academiae Scientiarum Hungaricae.

[B3398207] Sidhu A. K., Paliwal R,, Khanna V. (2012). Fauna of ecosystems of India–Western Himalaya, Ocassional paper.

[B3398231] Smith F. C. (1873). Untitled. Introduced by: "Mr. F. Smith exhibited a further collection of ants sent by Mr. G. A. James Rothney, from Calcutta". Transactions of the Entomological Society of London.

[B3501309] Wachkoo A. A. (2013). Taxonomy and species composition of Aenictinae, Cerapachyinae, Dorylinae, Formicinae and Ponerinae (Hymenoptera: Formicidae) from North-west Shivalik.

[B3398251] Wachkoo A. A. (2015). New status of the ant Camponotus mutilarius Emery, 1893 stat. nov. (Hymenoptera: Formicidae). Journal of Asia-Pacific Biodiversity.

[B3398261] Wachkoo A. A., Shah G. M., Jan U., Akbar S. A. (2016). A checklist of soldierflies (Diptera, Stratiomyidae) in India. Journal of Asia-Pacific Biodiversity.

